# Supplementary material for: Green Salad Intake Is Associated with Improved Oral Cancer Survival and Lower Soluble CD44 Levels
Source: Nutrients. 2021 Jan 26;13(2):372. doi: 10.3390/nu13020372 (PMC7911809; doi:10.3390/nu13020372)
Supplement: Supplementary file 1 [file nutrients-13-00372-s001.pdf]

Supplementary Table S1. log_2solCD44 and Protein Levels in Cancer Cases and Controls by Nutrition-related Variables and HPV status

Variable	Group				log_2[sol CD44] (with solCD44 in ng/ml)								Protein (mg/ml)							
	Cases		Controls		Case				Control				Case				Control			
					Unadjusted		Adjusted		Unadjusted		Adjusted		Unadjusted		Adjusted		Unadjusted		Adjusted	
	N	%	N	%	Mean	SEM	Mean	SEM	Mean	SEM	Mean	SEM	Mean	SEM	Mean	SEM	Mean	SEM	Mean	SEM
**All**	150	100	150	100	1.94	0.09	1.90	0.12	1.28	0.07	1.30	0.12	0.94	0.05	0.94	0.06	0.76	0.03	0.81	0.06
**Juices**	138	100	143	100																
<1/week or never	35	25.4	40	28.0	1.92	0.18	1.82	0.25	1.16	0.12	1.04	0.19	0.83	0.09	0.88	0.13	0.71	0.06	0.64	0.09
1-3/week or more	103	74.6	103	72.0	1.89	0.11	1.84	0.19	1.32	0.08	1.15	0.15	0.97	0.06	1.01	0.10	0.78	0.04	0.67	0.07
P value		0.621			0.874		0.947		0.321		0.473		0.211		0.290		0.329		0.745	
**Fruits**	143	100	142	100																
<1/week or never	34	23.8	22	15.5	1.72	0.16	1.53	0.26	1.31	0.13	1.24	0.22	0.89	0.08	0.88	0.14	0.87	0.09	0.78	0.10
1-3/week or more	109	76.2	120	84.5	1.94	0.11	1.87	0.18	1.23	0.08	1.10	0.14	0.93	0.06	0.97	0.09	0.73	0.03	0.64	0.06
P value		0.079			0.314		0.134		0.679		0.475		0.643		0.480		0.113		0.148	
**Potatoes**	137	100	143	100																
<1/week or never	21	15.3	50	35.0	2.21	0.26	2.19	0.29	1.29	0.13	1.20	0.17	1.05	0.10	1.08	0.16	0.78	0.06	0.71	0.08
1-3/week or more	116	84.7	93	65.0	1.80	0.10	1.75	0.18	1.25	0.08	1.14	0.14	0.89	0.05	0.95	0.10	0.75	0.04	0.68	0.07
P value		<.001			0.116		0.106		0.764		0.694		0.247		0.362		0.695		0.702	
**Carrots**	136	100	142	100																
<1/week or never	69	50.7	66	46.5	1.90	0.13	1.86	0.21	1.16	0.11	1.06	0.15	0.89	0.06	0.90	0.11	0.72	0.05	0.66	0.07
1-3/week or more	67	49.3	76	53.5	1.82	0.14	1.81	0.19	1.35	0.09	1.25	0.15	0.94	0.08	1.00	0.10	0.79	0.04	0.72	0.07
P value		0.478			0.649		0.791		0.189		0.195		0.564		0.315		0.262		0.392	
**Salads**	138	100	142	100																
<1/week or never	33	23.9	18	12.7	2.25	0.21	2.14	0.25	1.04	0.27	0.92	0.23	1.08	0.13	1.10	0.13	0.69	0.10	0.61	0.11
1-3/week or more	105	76.1	124	87.3	1.73	0.10	1.74	0.18	1.30	0.07	1.21	0.14	0.86	0.05	0.94	0.10	0.77	0.03	0.71	0.06
P value		0.015			0.014		0.081		0.355		0.187		0.122		0.177		0.399		0.371	
**Other vegetables**	138	100	146	100																
<1/week or never	23	16.7	20	13.7	2.11	0.24	2.06	0.31	1.13	0.25	1.00	0.23	1.15	0.17	1.20	0.16	0.80	0.10	0.67	0.11
1-3/week or more	115	83.3	126	86.3	1.83	0.10	1.76	0.18	1.32	0.07	1.24	0.14	0.87	0.05	0.91	0.09	0.76	0.04	0.72	0.07
P value		0.486			0.275		0.285		0.483		0.285		0.129		0.051		0.708		0.605	
**Salads/ Other vegs.**	139	100	146	100																
<1/week or never	14	10.1	7	4.8	2.12	0.34	1.96	0.35	1.00	0.66	0.83	0.35	1.25	0.27	1.22	0.19	0.78	0.22	0.65	0.16
1-3/week or more	125	89.9	139	95.2	1.83	0.10	1.77	0.18	1.31	0.07	1.24	0.14	0.88	0.04	0.93	0.09	0.77	0.03	0.72	0.07
P value		0.088			0.350		0.561		0.660		0.237		0.206		0.092		0.947		0.67	
**T-stage**	150	100																		
T3-4	87	58.0	--	--	2.07	0.13	2.08	0.18	--	--	--	--	0.97	0.07	1.01	0.09	--	--	--	--
T1-2	63	42.0	--	--	1.76	0.12	1.78	0.22	--	--	--	--	0.89	0.05	0.97	0.11	--	--	--	--
P value		--			0.088		0.151				--	--	0.401		0.721				--	--
**HPV status**	150	100																		
P16+	31	20.7	--	--	1.90	0.20	1.94	0.25	--	--	--	--	0.88	0.10	0.93	0.13	--	--	--	--
P16-	48	32.0	--	--	1.99	0.16	1.86	0.22	--	--	--	--	0.88	0.08	0.92	0.11	--	--	--	--
NA	71	47.3	--	--	1.93	0.13	2.00	0.20	--	--	--	--	1.00	0.07	1.11	0.10	--	--	--	--
P value	-	--			0.946		0.829						0.47		0.174					

SEM: standard error of the mean. Comparison of proportions between cases and controls by the chi-square test.

Unadjusted means by nutrition-related variable, t-stage or HPV status, separately within cases and controls, were compared using the Student's t-test or ANOVA.

Adjusted means were estimated and compared from fitting multivariable regression models including a nutrition-related variable, adjustment for age (<60, 60 or more), race (black, non-black/missing), ethnicity (Hispanic, non-Hispanic), gender (male, female), smoking history (never, ever), drinking habits (non-drinker/mild, moderate/heavy), SES (low, high), oral heath score (good, poor/fair/missing), teeth removed (5 or less, 6 or more/All/miss), and gargle (good, poor/fair/missing), and only for cases, additional adjustment for disease T-stage and HPV status.

Of note, comparing to controls, cases had statistically significant higher levels of CD44 (log2solCD44 estimated mean 1.90 vs. 1.30, p<.0001, which corresponds to CD44 values 3.73 vs. 2.46, respectively) and of protein (estimated mean 0.94 vs. 0.81, p=0.0243) based on multivariable models including group and adjustment for the 10 common variables among cases and controls.
